# Efficient CRISPR/Cas9-mediated biallelic gene disruption and site-specific knockin after rapid selection of highly active sgRNAs in pigs

**DOI:** 10.1038/srep13348

**Published:** 2015-08-21

**Authors:** Xianlong Wang, Jinwei Zhou, Chunwei Cao, Jiaojiao Huang, Tang Hai, Yanfang Wang, Qiantao Zheng, Hongyong Zhang, Guosong Qin, Xiangnan Miao, Hongmei Wang, Suizhong Cao, Qi Zhou, Jianguo Zhao

**Affiliations:** 1 State Key Laboratory of Reproductive Biology, Institute of Zoology, Chinese Academy of Sciences, Beijing 100101, China; 2 College of Veterinary Medicine, Sichuan Agriculture University, Ya’an, Sichuan 625014, China; 3 University of Chinese Academy of Sciences, Beijing 100049, China; 4 Institute of Animal Sciences, Chinese Academy of Agricultural Sciences, Beijing 100193, China

## Abstract

Genetic engineering in livestock was greatly enhanced by the emergence of clustered regularly interspaced short palindromic repeats (CRISPR)/CRISPR-associated 9 (Cas9), which can be programmed with a single-guide RNA (sgRNA) to generate site-specific DNA breaks. However, the uncertainties caused by wide variations in sgRNA activity impede the utility of this system in generating genetically modified pigs. Here, we described a single blastocyst genotyping system to provide a simple and rapid solution to evaluate and compare the sgRNA efficiency at inducing indel mutations for a given gene locus. Assessment of sgRNA mutagenesis efficiencies can be achieved within 10 days from the design of the sgRNA. The most effective sgRNA selected by this system was successfully used to induce site-specific insertion through homology-directed repair at a frequency exceeding 13%. Additionally, the highly efficient gene deletion via the selected sgRNA was confirmed in pig fibroblast cells, which could serve as donor cells for somatic cell nuclear transfer. We further showed that direct cytoplasmic injection of Cas9 mRNA and the favorable sgRNA into zygotes could generate biallelic knockout piglets with an efficiency of up to 100%. Thus, our method considerably reduces the uncertainties and expands the practical possibilities of CRISPR/Cas9-mediated genome engineering in pigs.

Pigs are an important source of food and nutrition in humans and are widely used to study a variety of human diseases. The efficient and precise genetic modification of pigs would facilitate the generation of tailored disease models and strains with valuable agricultural traits^1^,^2^. However, despite the large number of available techniques, such as pronuclear injection^3^, sperm-mediated transfection^4^,^5^, oocyte transduction^6^, and intracytoplasmic sperm injection (ICSI)-mediated transgenesis^7^, the generation of a genetically engineered pig by homologous recombination remains a relatively time-consuming procedure. Somatic cell nuclear transfer (SCNT) has facilitated the ability to make genome modified pigs by circumventing most of the shortcomings of above techniques. However, the SCNT has low efficiency and has been hampered by establishment of cell lines with the desired genetic modification due to a lack of available germ line-competent pluripotent stem cells^8^,^9^. Several genome-engineering techniques have been developed for guiding nucleases to induce site-specific double-strand breaks (DSBs) in the genome, making it possible to efficiently generate genetically modified pigs^10^,^11^,^12^,^13^,^14^,^15^,^16^.

The recently developed Type II bacterial clustered regularly interspaced short palindromic repeats (CRISPR)/CRISPR-associated (Cas) system has been recently developed and adapted to genome editing^10^,^11^. This system requires a 20-nucleotide guide sequence contained within an associated CRISPR RNA (crRNA) transcript, a trans-activating crRNA (tracrRNA) partially complementary to the crRNA, and a Cas endonuclease to catalyze DNA cleavage^17^. The Cas9 endonuclease from the *Streptococcus pyogenes* type II CRISPR/Cas system can be engineered to produce targeted genome modifications in a sequence-specific manner by providing a synthetic single-guide RNA (sgRNA) consisting of a fusion of crRNA and tracrRNA^18^. This CRISPR/Cas9 system has been successfully adapted to generate genetically modified animals, including mice^19^, rats^20^, zebrafish^21^, frogs^22^, fruit flies^23^, monkeys^24^, and livestock^25^,^26^,^27^,^28^.

Recently, the CRISPR/Cas9 system was demonstrated to efficiently generate biallelic knockout pigs through a direct cytoplasmic injection of Cas9 mRNA and sgRNA into pig zygotes^25^. This indicated that the CRISPR/Cas9 system shows potential in complex pig genome engineering. However, given the lengthy gestation period and the high cost of housing, it is a challenge in pigs to confirm the presence of the indel mutation in the target sequence of modified pig genomes using chromatin samples from fetuses or newborn piglets after the completion of an actual experiment. Moreover, intensive labor and numerous sows are required to obtain a sufficient number of *in vivo*-derived zygotes. Therefore, an optimized CRISPR/Cas9-based genome engineering pig system can maximize the efficiency of genetic modifications.

Although sgRNA activity can be quite high, there is significant variability among sgRNAs in their ability to produce null alleles and sgRNA targeting efficiency varies significantly between loci and even between target sites within the same locus^29^,^30^,^31^. For precise genetic modification (knockin or base substitution), the targeting efficiency of the sgRNA is the most critical factor than general gene deletion^32^. Thus, selecting the most effective sgRNAs for a particular gene locus would greatly expand the utility of a porcine CRISPR/Cas9 system. In the present study, we rapidly estimated the sgRNA efficiency at inducing indel mutations by single blastocyst genotyping. Then, the most favorable sgRNA was verified by mediating knock-in in embryos and generating knockout pigs. Our method considerably reduces the uncertainties and expands the practical possibilities of genome engineering in livestock.

## Results

### Design and construction of CRISPR

**Figure 1:**
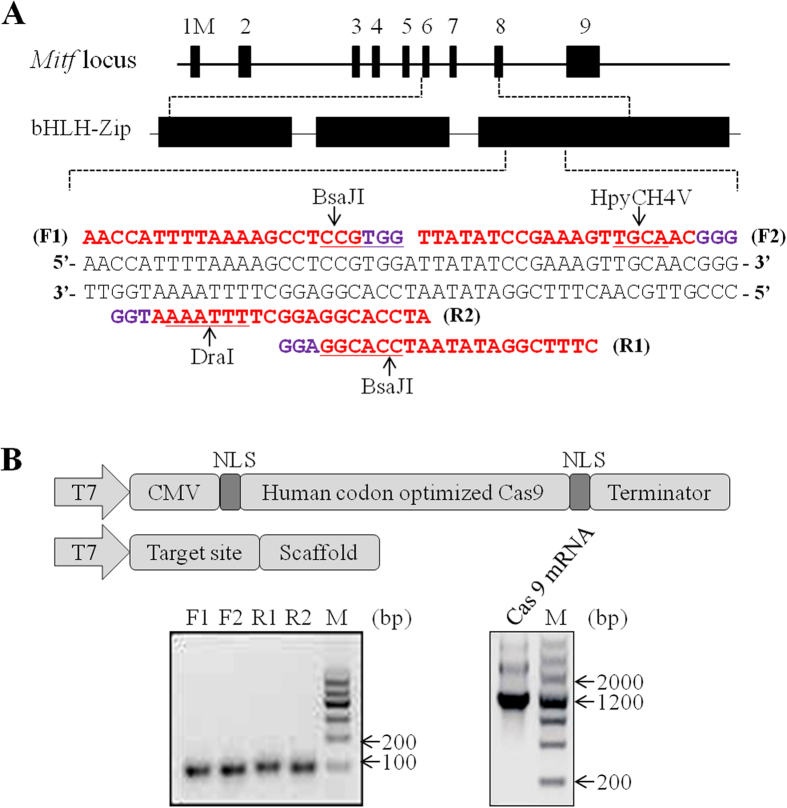
Design and construction of CRISPR. (**A**) Schematic of the Cas9/sgRNA-targeting sites in pig *mitf*loci. Exons are shown as black boxes. The sgRNA-targeting sequence is labeled in red, and the protospacer-adjacent motif (PAM) sequence is labeled in purple. The different sgRNAs are marked as F1, F2, R1 and R2, respectively. The restriction sites at the target regions are underlined. Restriction enzymes used for RFLP analysis are shown. (**B**) Top: schematic diagram of the templates for *in vitro* transcription used to generate the Cas9 mRNA and sgRNA. Bottom: assessing the quality of sgRNAs and Cas9 mRNA by gel electrophoresis. Electrophoresis of sgRNAs yields a single band approximately 100 bp. Electrophoresis of Cas9 mRNA yields 2–3 bands due to persistent secondary structure. The quality of mRNA is good if discrete bands are visible.

MITF protein is a master regulator of melanocyte development and an important oncogene in melanoma^33^. Mutations in the human *mitf* gene have been found in patients with the hypopigmentation and deafness syndromes, Waardenburg (WS) and Tietz (TS)^34^. Recently, numerous pig models of human diseases have been developed using gene targeting approach owing to pig sharing more physiological similarities with humans. It prompts us to generate *mitf* genes knockout pigs to model human WS and TS syndromes. We designed four different sgRNAs (F1, F2, R1 and R2) that target 47 bp regions of exon 8 of the pig *mitf* gene (Fig. 1A), which is a part of the basic helix-loop-helix leucine zipper (bHLH-Zip) domain sequence and is essential for MITF DNA-binding activity^35^. The sgRNA target sequence (20 nt) did not cross-react with any other sites in the pig genome and was followed by an NGG protospacer adjacent motif (PAM), which is necessary for Cas9 cleavage.

Cas9 mRNA was generated by the *in vitro* transcription of a linearized and T7 promoter-driven pXT7-hCas9 plasmid template, which included a human-codon-optimized version of Cas9 cDNA and nuclear localization signals (NLSs) at both ends of Cas9 (Fig. 1B). The T7-sgRNA PCR products for the *in vitro* transcription of sgRNA were obtained as described in the Material & Methods section. Only good-quality, purified sgRNAs and Cas9 mRNA (as assessed by gel electrophoresis) were used for oocyte injections (Fig. 1B).

### Rapidly selecting the most effective sgRNAs by single blastocyst genotyping

**Figure 2:**
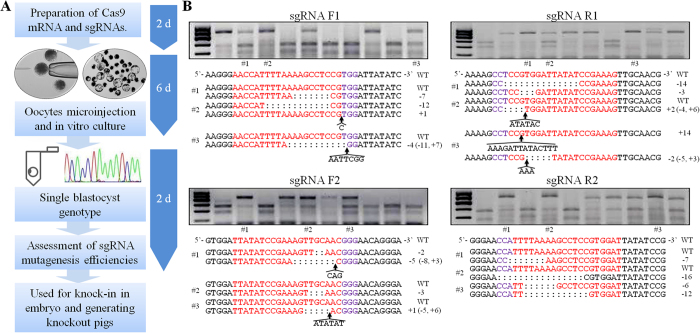
Rapidly selecting most effective sgRNAs by single blastocyst genotyping. (**A**) Overview and timeline of the experiment. Beginning with experimental design, assessment of sgRNA mutagenesis efficiencies can be achieved within 10 days. (**B**) Genotyping of single blastocyst derived from Cas9 mRNA and sgRNA-injected parthenogenetic oocytes by RFLP and Sanger sequence analyses. Representative RFLP agarose gel electrophoresis showing PCR product of target region derived from 10 individual blastocysts digested with different restriction enzymes. The F1, F2, R1 and R2 sgRNA mutagenesis efficiencies were assessed by BsaJI, HpyCH4V, BsaJI and DraI, respectively. In each RFLP assay, some PCR products were sequenced to confirm the mutations at the target sites. The numbers on the right show the type of mutation and how many nucleotides are involved, with “−” and “+” indicating deletion or insertion of the given number of nucleotides, respectively. The sgRNA sequence is labeled in red, and the PAM sequence is labeled in purple. Deleted bases are marked with colons, and arrows indicate the sites of inserted bases, which are listed under the mutant alleles.

The lack of a simple platform to unbiasedly evaluate the efficacies of sgRNA creates uncertainties and restricts the ability to modify the pig genome. Toward this end, we developed an experimental system to rapidly select the most favorable sgRNA for a specific gene locus based on single-blastocyst genotyping. An overview of the experimental process with approximate timings is shown in Fig. 2A.

To compare the mutagenesis efficiencies of different sgRNAs, approximately 2–10 pL of RNA mixture (containing 125 ng/μL of Cas9 mRNA and 12.5 ng/μL of individual sgRNA) were microinjected into the cytoplasm of mature MII pig oocytes. After parthenogenetic activation, oocytes were cultured to the blastocyst stage. The *in vitro* blastocyst rate of oocytes injected with Cas9 mRNA/sgRNA (F1, 27.68%; F2, 25.99%; R1, 26.61%; R2, 25.44%; respectively) and oocytes injected with water (28.16%) were normal and comparable with each other (Table 1), suggesting that the Cas9 mRNA and sgRNA had low or no toxicity for early pig embryonic development. A single blastocyst was randomly selected and lysed for genotyping analysis.

PCR products, including the target site, were amplified and analyzed by restriction fragment length polymorphism (RFLP) for identification of the mutations (Fig. 2B). A failure of the restriction enzyme digestion suggested the occurrence of DNA sequence mutations in the target regions. Some of the non-digested PCR products were sequenced and aligned to reference sequences, which confirmed that the losses of the respective restriction sites were due to mutations at the target sites (Fig. 2B).

The RFLP analysis showed that all four sgRNAs could induce indel mutations in the target region but with different mutagenesis efficiencies. As summarized in Table 1, the sgRNAs generated the mutant embryos at approximately 50–80% efficiencies. Among the tested sgRNAs, the R1 sgRNA produced approximately 12% monoallelic and 69% biallelic mutant embryos, suggesting that R1 was the most favorable sgRNA for the CRISPR/Cas9 system in this target region.

### Highly efficient R1 sgRNA-mediated knock-in in porcine embryo

**Figure 3:**
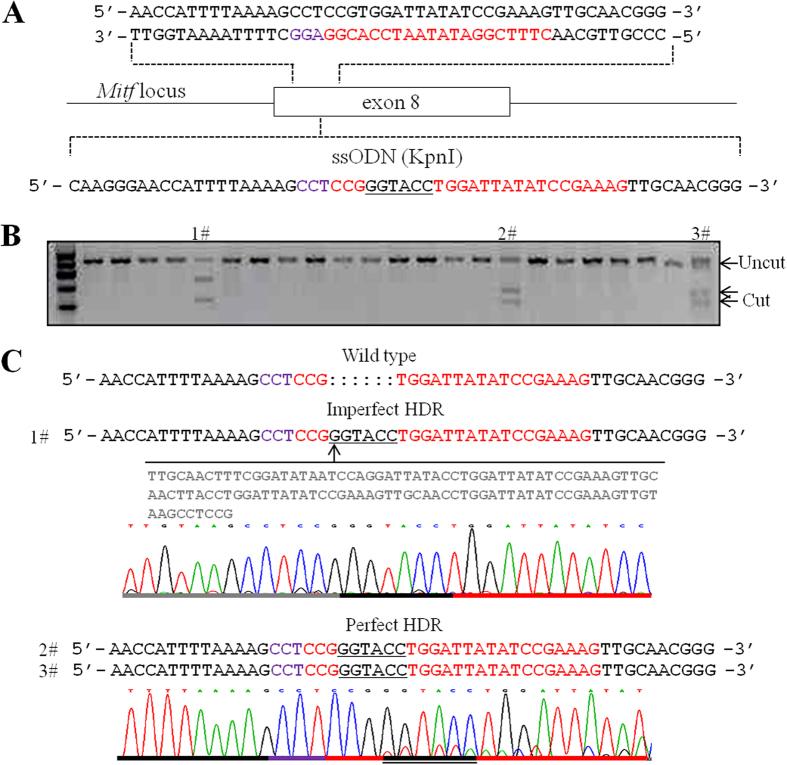
R1 sgRNA-induced site-specific insertion with ssODN through HDR.A. (**A**) schematic of the targeting site with the ssODN sequence used to introduce an exogenous KpnI sequence (underlined). The sgRNA sequence is labeled in red, and the PAM sequence is labeled in purple. (**B**) RFLP agarose gel electrophoresis showing PCR product of target region derived from individual blastocyst digested with KpnI restriction enzymes. Three out of 23 blastocysts demonstrated site-specific KpnI sequence insertion through HDR. (**C**) Sequence analysis of the three blastocysts at the target site. Two blastocysts showed precise HDR-based addition of the KpnI sequence. One blastocyst showed precise addition at the 3′ end, and 117 bp indels were noted at the 5′ side of the modification site. The sgRNA sequence is labeled in red, and the PAM sequence is labeled in purple. KpnI sequence is underlined.

The DSBs mediated by CRISPR/Cas9 can stimulate a homologous recombination in the presence of a DNA donor with the appropriate homology arms. Recent works have demonstrated that single-stranded DNA oligonucleotides (ssODNs) can be used as substitutes for conventional plasmid-based targeting vectors as donor templates for homology-directed repair (HDR)^36^. Because of its high mutagenesis efficiency, we hypothesized that R1 sgRNA could assist in HDR. Hence, we co-injected Cas9 mRNA, sgRNA and ssODN containing 6 bp KpnI restriction site flanked by 26 bps homologous sequences on each side into mature MII pig oocytes (Fig. 3A). After parthenogenetic activation, oocytes were cultured to the blastocyst stage and single blastocysts were picked for genotyping. In these experiments, the RFLP assays, as shown in Fig. 3B, identified 3 out of 23 R1 sgRNA-injected blastocysts carrying the KpnI site at the target locus, indicating R1 sgRNA yielded an HDR efficiency as high as 13.04% with ssODN at a concentration of 80 ng/μL. Subsequent sequence analyses indicated two precise KpnI site insertions, which demonstrated successful targeted restriction site insertions by R1 sgRNA-mediated HDR in pig embryos (Fig. 3B). Another insertion showed a precise addition at the 3′ end, whereas 117 bps indels were noted at the 5′ side of the modification site (Fig. 3C). In addition, we failed to detect HDR induced by F2 sgRNA or R1 sgRNA with 10 ng/μL ssODN (Table 2), suggesting that CRISPR/Cas9 and ssODN-mediated HDR were highly dependent on the targeting efficiency and the ssODN concentration.

### Highly efficient R1 sgRNA-mediated gene targeting in porcine fibroblasts

**Figure 4:**
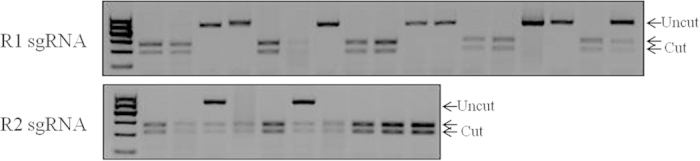
Genotyping of single fibroblast colonies derived from FACS sorting based on the expression of EGFP fluorescence by RFLP analysis. Agarose gel electrophoresis showing PCR product of target region digested with different restriction enzymes. The R1 and R2 sgRNA mutagenesis efficiencies were assessed by BsaJI and DraI, respectively.

The dominant strategy for generating transgenic pigs is to first genetically modify fibroblasts and then conduct SCNT. To determine whether the selected sgRNA by mRNA injection into PA-derived blastocysts can induce highly efficient mutations in somatic cells, we investigated the mutagenesis efficiencies of different sgRNAs in porcine primary fibroblasts. The plasmids expressing Cas9 and R1 or R2 sgRNA were transfected into fibroblasts, and transfected single cells were sorted and cultured in 96-well plates. Of 17 colonies obtained by R1 sgRNA transfections, eight carried mutations in the target sequence and seven had biallelic mutations. However, of the 10 colonies obtained by R2 sgRNA transfection, only two colonies carried mutations in the target sequence, and none had biallelic mutations (Fig. 4). The results (summarized in Table 3) demonstrated that the mutagenesis efficiencies of sgRNAs in fibroblasts were comparative with those in single blastocyst assays, suggesting that the mutagenesis efficiencies of a given sgRNA were consistent in both embryo and somatic cells.

### Generation of Mitf knockout pigs by zygote injection of R1 sgRNA and Cas9 mRNA

**Figure 5:**
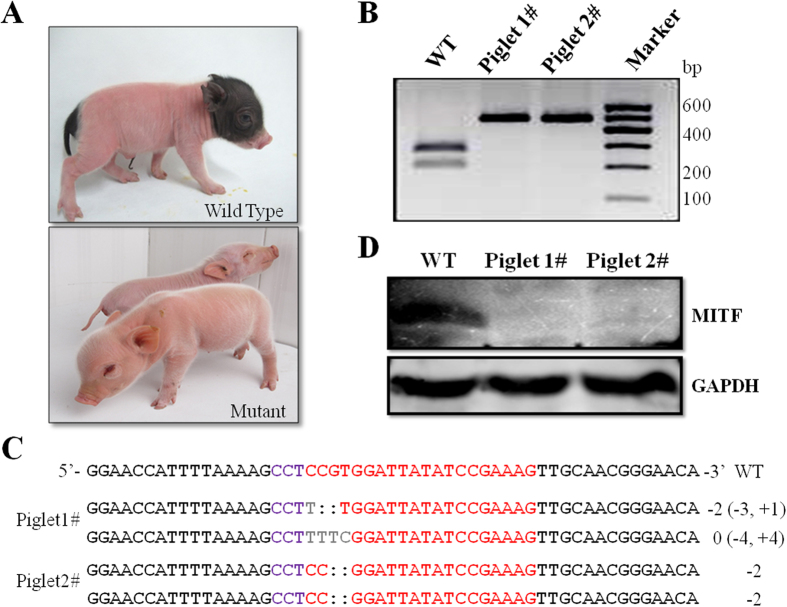
Generation of Mitf knockout pigs by zygote injection of R1 sgRNA and Cas9 mRNA. (**A**) Newborn wild type pig (upper panel) and piglets carrying *Mitf* gene mutation (bottom panel). The photographs were taken by the author, Xianlong Wang. (**B**) RFLP agarose gel electrophoresis showing PCR product of target region derived from two piglets digested with BsaJI restriction enzymes. (**C**) Sanger sequencing of the targeting site in mutant pigs. The wild-type (WT) sequence is shown at the top, where the sgRNA sequence is labeled in red and the PAM sequence in purple. The numbers on the right show the type of mutation and how many nucleotides are involved, with “−” and “+” indicating deletion or insertion of the given number of nucleotides, respectively. Deleted bases are marked with colons, and inserted bases are gray. (**D**) Western blot for MITF protein expression in the skin tissues of tail from wild type and mutant piglets. GAPDH was used as a loading control.

The ultimate aim of this study was to efficiently generate genetically modified pigs through the direct cytoplasmic injections of Cas9 mRNA and sgRNA into zygotes. Thus, we next transferred the Cas9 mRNA and R1 sgRNA-injected zygotes into surrogate pigs to produce piglets. A total of 40 embryos were delivered to 3 surrogates, and one pregnancy was established (Table 4). Two live-born piglets were obtained and showed the white coat-color phenotype over its entire body (Fig. 5A); the wild-type pigs exhibited pigment deposition at the two ends of the body (Fig. 5A). RFLP (Fig. 5B) and sequence analysis (Fig. 5C) assays showed that the piglets were all genotyped as bi-allelic mutations, suggesting that R1 sgRNA could efficiently facilitate the CRISPR/Cas9 system to generate Mitf knockout pigs. The skin tissues of the tail were dissected from the mutant piglets, and Western blot analysis was performed to confirm the disruption of MITF in these pigs. Compared with wild-type piglet, MITF was completely absent in the two mutant piglets (Fig. 5D). Moreover, the genomic DNA isolated from the two mutant piglets were used to perform off-target analyses. The fragments around the potential off-target loci were amplified and sequenced. No unwanted mutations occurred at these genomic sites of the two mutant piglets (Supplemental Table S2 and Figure S1).

## Discussion

The CRISPR/Cas9 system could efficiently generate genetically modified pigs for agricultural and biomedical purposes. However, prior studies have suggested that there are significant variabilities among sgRNAs in their ability to produce null alleles^29^,^30^,^31^. The herein described single blastocyst genotyping system provides a simple method to evaluate and compare the mutagenesis efficiency of different sgRNAs for a given gene locus. Using this system, the most favorable sgRNA rapidly and inexpensively creates genetically modified living organisms, especially large livestock animals, such as pigs.

The chromatin environment around the target sites and sgRNAs sequence features have recently been identified as major factors governing the on-target efficacy of the CRISPR/Cas9 system^37^,^38^. The former affected the ability of Cas9 to find the PAM and bind DNA with the seed region of the sgRNA. In this study, it was unlikely that the variability of sgRNAs mutagenesis efficiencies were due to differences in local chromatin structures because the four studied sgRNAs target sites were in a 47 bp region. Rather, the sgRNA sequence itself likely affected the mutagenesis efficiencies. Indeed, Doench *et al.*, quantitatively assayed the activity of thousands of sgRNAs to uncover sequence features that modulate the ability of Cas9 to bind DNA, cleave the target site and produce a null allele, and found sequence features that are predictive of sgRNA activity^29^. For example, the high-activity sgRNA strongly preferred guanine in sequence position 20 and the nucleotide immediately adjacent to the PAM, whereas cytosine was strongly unfavorable. In agreement with this observation, in the present study, the R1 sgRNA with high activity was characterized by guanine at position 20, and the F2 sgRNA with a relatively low activitywas characterized by cytosineat this position. However, we failed to meet the sequence features in position 16 and the nucleotide sequence of the PAM. Additional studies utilizing a combination of bioinformatics techniques and actual experimental systems will more objectively provide an assessment of the mutagenesis efficiencies of different sgRNAs.

Sakurai *et al.*^39^ also described a single blastocyst assay for detecting mutations introduced by the CRISPR/Cas9 system based on whole genome amplification (WGA) technology. Although WGA enables multiple analyses of genomic DNA, it increases the duration and costs and potentially introduces unwanted mutations. A target proto spacer DNA reporter system was recently developed to test the efficiency of sgRNA targeting in *in vitro* cultured cells by artificially locating sgRNA target sites on extra-chromosomal plasmids^40^. Consequently, the application of this system is limited because does not mimic the natural chromatin environment around the target sites and requires a steady transfection efficiency. Zhou *et al.* reported that the simultaneous use of dual sgRNAs to target an individual gene significantly improved the Cas9-mediated genome targeting with a bi-allelic modification efficiency of up to 78%^41^. What is noteworthy is that more sgRNAs would give rise to higher rates of off-target mutagenesis. Our described single-blastocyst genotyping system overcame the above defects, but should be used with caution. The blastocysts tested in this study were obtained from parthenogenetically activated oocytes. Several studies have demonstrated that parthenogenetic and fertilized embryos present different chromatin environmentsand DNA methylation patterns around the imprinting control regions^42^,^43^,^44^. This indicates that the mutagenesis efficiencies of different sgRNAs for an imprinting gene locus might be not comparable.

As described above, the ultimate aim of this study was to efficiently generate genetically modified pigs. To verify the effectiveness of our system, the selected R1 sgRNA was used to mediate oligonucleotide knock-in in porcine embryos and to generate the gene knockout piglets. The most efficient R1 sgRNA produced 13.04% knock-in rates. Additionally, with respect to precise integrations at 8.7%, the rate of HDR was still higher. To the best of our knowledge, this study is the first to demonstrate ssODN knock-in by a CRISPR/Cas9 system in pig. In targeting the gene locus *in vivo* via zygote injection, R1 sgRNA improved the Cas9-mediated genome targeting with a bi-allelic modification efficiency up to 100%. This suggests that our system could maximize sgRNA activity.

In conclusion, we developed a single blastocyst genotyping system to provide a simple and rapid method to evaluate and compare the efficiency of sgRNAs at inducing indel mutations introduced by the CRISPR/Cas9 system for a given gene locus. The efficiency of the selected sgRNA was confirmed by the success of site-specific knock-ins, efficient gene targeting in porcine fibroblasts and the generation of a bi-allelic gene targeting pig model. Our method considerably reduced the uncertainties and expanded the practical possibilities of CRISPR/Cas9-mediated genome engineering in pigs.

## Material and Methods

### Chemical and Reagents

Unless otherwise stated, all chemicals were purchased from Sigma (St. Louis, MO).

### Ethics statement

All experiments involving animals were conducted according to the Guidelines for the Care and Use of Laboratory Animals established by the Beijing Association for Laboratory Animal Science and approved under the Animal Ethics Committee of Institute of Zoology, Chinese Academy of Sciences.

### Recovery of *in-vivo*-derived zygotes

*In vivo* zygotes were retrieved surgically from natural mating gilts. The animals were monitored for estrus twice daily by observing their response to a mature boar, reddening of the vulva, and vaginal mucus secretions. The gilts were mated immediately with a mature boar following detection of estrus. After 24 h, surgeries were performed to excise the urogenital tract under general anesthesia. The number of zygotes in the oviduct was estimated by examining the ovaries and counting the ruptured follicles. Zygotes were flushed from the oviducts by using a 10-ml syringe twice with prewarmed PBS containing 1% (vol/vol) polyvinyl alcohol (PVA). Recovered zygotes were washed and ready for cytoplasmic microinjection of RNAs.

### Production of Cas9 mRNA and sgRNA

The pXT7-hCas9 plasmid for the *in vitro* transcription of humanized Cas9 mRNA was obtained from the China Zebrafish Resource Center. The vector was linearized by XbaI (New England Biolabs, Inc., MA, USA) digestion. Capped mRNA was synthesized using Ambion mMESSAGE mMACHINE mRNA transcription synthesis kits (Life Technologies) and purified using the RNAclean Kit (Tiangen, Beijing, China).

Guide RNAs were designed using an online tool provided by Feng Zhang’s Laboratory at the MIT/BROAD Institute (http://crispr.mit.edu/). The T7 promoter was added to the sgRNA template by PCR amplification using primers (Supplemental Tables S1). The T7-sgRNA PCR product was gel-purified and used as the *in vitro* transcription template using a MEGAshortscript T7 kit (Life Technologies, US). The sgRNAs were subsequently purified using a mirVana™ miRNA Isolation Kit (Life Technologies, US) and eluted into RNase-free water. The qualities of the RNAs were checked by gel electrophoresis.

### Oocyte collection and *in vitro* maturation

Porcine ovaries were collected from prepubertal gilts at a local slaughterhouse and transported to the laboratory in a vacuum flask (30–35 °C) containing sterile physiological saline within 2–3 h of collection. Follicles between 2 mm and 6 mm indiameter were aspirated with an 18-gauge needle attached to a 10-mL syringe. Cumulus-oocyte complexes (COCs) within the follicular fluid were allowed to settle by gravity at 37 °C. The COCs were rinsed three times in HEPES-buffered Tyrode medium containing 0.01% PVA in an incubator at 37 °C. COCs with multiple layers of intact cumulus cells and uniform ooplasm were selected for IVM. After washing three times in IVM medium, a group of 70–80 COCs were placed into wells of four-well cell culture plates (Nunc, Roskilde, Denmark) containing 500 μL *in vitro* maturation medium and 350 μL mineral oil per well. The COCs were cultured for 42–44 h at 38.5 °C and 5% CO2 in air (100% humidity). Matured COCs were then vortexed in 0.1% hyaluronidase in HEPES-buffered Tyrode medium containing 0.01% PVA for 4 min to remove the cumulus cells. Only the matured oocytes having an extruded first polar body (PB) with uniform cytoplasm were further used.

### Preparation of single-stranded DNA oligonucleotides (ssODN)

The ssODN donor templates (Supplemental Tables S1) were synthesized as normal oligonucleotides and purified by PAGE (Life Technologies, US). ssODNs were diluted with RNase free water to 100 μM, divided into aliquots and stored at −20 °C.

### Cytoplasmic microinjection of RNAs

Cas9 mRNA, gRNA or ssODN was injected into the cytoplasm of matured oocytes or zygotes using a FemtoJet microinjector (Eppendorf; Hamburg, Germany) according to previous reports. To select the highest efficient sgRNA, *in vitro*-produced parthenogenetic embryos were used in a preliminary experiment. Parthenogenetic activation of the injected oocytes wasaccomplished with two direct current pulses (1-sec intervals) of 1.2 kV/cm for 30 microseconds provided by a BTX Electro-cell Manipulator 200 (BTX, San Diego, CA) in fusion medium. Then, the activated oocytes were cultured to blastocyst stage for genotyping in PZM3 medium for 144 hours at 38.5 °C and 5% CO_2_ in humidified air.

### Single blastocyst genotyping

Crude DNA derived from a single blastocyst was prepared according to the method described by Sakurai *et al.*^39^ with some modifications. Briefly, under a stereomicroscope (Leica, Germany), 0.5 μL of PBS (pH 7.4) containing 1 blastocyst was transferred to the wall near the bottom of a 0.2-mL PCR tube using a micropipette. Thereafter, 9 μL of lysis buffer and 0.5 μL PCR-grade Proteinase K (both from TIANcombi DNA Lyse&Amp PCR Kit, Tiangen Biotech, Beijing, China) was gently added to each tube. After a brief centrifugation, 0.5 μL of mineral oil wasadded to the mixture to prevent evaporation. To lyse the blastocyst, each PCR tube was incubated at 56 °C for 10 min and then at 95 °C for 5 min. The resulting crude DNA solution was stored at −20 °C until use.

For blastocyst genotyping, the amplifications were obtained by two rounds of PCR. A first round of PCR was amplified in a 20 μL volume containing 10 μL of Phusion® High-Fidelity PCR Master Mix (New England Biolabs, US), 0.5 μM forward and reverse primers, and 5 μL of crude DNA solution. The PCR cycling times were 98 °C for 2 min followed by 20 cycles using the following conditions: denaturation for 10 s at 98 °C, annealing for 20 s at 62 °C, and extension for 30 s at 72 °C. Amplification was completed with a final extension at 72 °C for 5 min. A 2 μL aliquot of the first round reaction was added to the second round PCR mixture to a total volume of 20 μL containing 10 μL of Phusion® High-Fidelity PCR Master Mix and 0.5 μM forward and reverse primers. The amplification was carried out for 40 cycles using the same procedure as that for the first round of PCR. The sequences of the PCR primers are shown in Supplemental Table S1.

The identity of each PCR product was confirmed by restriction fragment length polymorphism (RFLP) analysis. Ten microliters of PCR product was digested with BsaJI, HpyCH4V or DraI (New England Biolabs, US), respectively. The reaction products were separated by 2% agarose gel electrophoresis in the presence of ethidium bromide solution, and visualized with a UV transilluminator (UVP, Upland, CA).

### Embryo transfer

The surviving embryos were transferred into the oviduct of recipient gilts on the day or 1 day after the onset of estrusfollowing a mid-line laparotomy under general anesthesia. Pregnancy was diagnosed after 28 days, and then each pig was checked regularly at 2-week intervals by ultrasound examination. All of the microinjected piglets were delivered by natural birth.

### Design and construction of CRISPR/Cas9 plasmids

To obtain CRISPR/Cas9 constructs targeting genomic sequences, the pX330 vector, created by the laboratory of Dr. Feng Zhang and obtained from Addgene (Plasmid 42230), was used. The R1 and R2 sgRNA genome targeting sequences were cloned into the pX330 vector as previously described^45^.

### Cell culture and transfection

Porcine primary fetal fibroblast cells (FFCs) were cultured and transfected as previously described^46^. The pCAG-GFP plasmid was co-transfected with pX330 plasmid to be used as an indicator for FACS sorting. Forty-eight hours after transfection, cells were subjected to FACS sorting based on the expression of EGFP fluorescence. Single cells were plated in each well of 96-well plates and cultured for approximately 10 days in cell culture medium supplemented with 2.5 ng/mL basic fibroblast growth factor (Sigma, St. Louis, MO). The medium was replaced every 4 days. Confluent cell colonies were propagated and genotyped by RFLP assay and sequencing.

### Sanger sequencing of mutated sites

Mutagenesis at the targeted site was assessed by PCR-based assays. The PCR products were cloned into pMD18-T vectors (Takara) and transformed into *Ε.coli* DH5-α competent cells (Tiangen). Fifteen positive colonies were picked and sequenced. Mutations were identified by alignment of the sequenced alleles to the wild-type alleles.

### Western blot analysis

The skin tissues of the tail were obtained from wild type or mutant pigs. Twenty to thirty milligrams of skin tissues were homogenized in RIPA buffer (Thermo Fisher Scientific, Waltham, MA, USA) and then centrifuged at 600 × g for 15 min to pellet any insoluble material, and 50 μg of protein was subjected to Western blot analysis. The MITF antibody (ab12039) was purchased from Abcam (Cambridge, MA, USA), and a concentration of 1:1000 was used to detect the expression of MITF in skin tissues. GAPDH was used as housekeeping genes to confirm equal sample loading. The GAPDH antibody (cw0101) was purchased from Cwbiotech (Beijing, China).

### Off-target analysis

A total of 39 potential R1 sgRNA off-target sites in the pig genome were predicted by the CRISPR design tool (http://crispr.mit.edu/). The first ten hits, termed off-target 01 through off-target 10 sites (Supplemental Table S2) were PCR amplified and subjected to TA cloning, respectively. Fifteen positive colonies for each potential off-target site were randomly selected and sequenced. The primers for amplifying the off-target sites are listed in Supplemental Table S1.

## Additional Information

**How to cite this article**: Wang, X. *et al.* Efficient CRISPR/Cas9-mediated biallelic gene disruption and site-specific knockin after rapid selection of highly active sgRNAs in pigs. *Sci. Rep.* **5**, 13348; doi: 10.1038/srep13348 (2015).

## Supplementary Material

Supplementary Information

## Figures and Tables

**Table 1:** Efficiency of CRISPR/Cas9-mediated gene targeting depends on sgRNAs sequence features.

**sgRNA**	**Blastocysts/injected oocytes (blastocyst rate)**	**No. of tested blastocysts**	**Monoallelic mutants (% of tested)**	**Biallelic mutants (% of tested)**
**Water**	**29/103 (28.16%)**	\	\	\
F1	106/383 (27.68%)	41	21 (51.22)	4 (9.76)
F2	98/377 (25.99%)	30	6 (20.00)	9 (30.00)
R1	95/357 (26.61%)	55	7 (12.73)	38 (69.09)
R2	87/342 (25.44%)	38	22 (57.89)	4 (10.53)

Comparison of *in vitro* development of CRISPR/Cas9-injected oocytes and mutagenesis efficiency between different sgRNAs.

**Table 2:** Summary of CRISPR/Cas9-induced site-specific insertion with ssODN through HDR.

**Injected components**	**No. of tested blastocysts**	**HDR blastocysts (% of tested)**	**Precise HDR (% of tested)**
Cas9 mRNA + R1 sgRNA + ssODN (80 ng/μL)	23	3 (13.04)	2 (8.70)
Cas9 mRNA + R1 sgRNA + ssODN (10 ng/μL)	20	0	0
Cas9 mRNA + F2 sgRNA + ssODN (80 ng/μL)	19	0	0

Different pools as indicated were coinjected into mature MII pig oocytes. After parthenogenetic activation, oocytes were cultured to the blastocyst stage and a single blastocyst was selected for genotyping by RFLP and Sanger sequencing assays.

**Table 3:** Summary of CRISPR/Cas-mediated gene targeting in porcine primary fibroblasts.

**sgRNA**	**No. of tested colonies**	**Monoallelic mutants (% of tested)**	**Biallelic mutants (% of tested)**
R1	17	1 (5.88)	7 (41.18)
R2	10	2 (20.00)	0 (0)

**Table 4:** Summary of generated Mitf mutant pigs via zygote injection of Cas9 mRNA and R1 sgRNA.

	**Injected zygotes**	**Transferred zygotes**	**Surrogate pregnancy**	**Newborns**	**Mutants**	**Biallelic mutants (% of newborns)**
Experiment 1	17	15	No	0	0	0
Experiment 2	16	12	No	0	0	0
Experiment 3	17	13	Yes	2	2	2 (100%)

Surviving embryos were transferred into the oviduct of recipient gilts on the day or 1 day after the onset of estrus. Piglets were delivered by natural birth.

## References

[b1] Prather R. S. Pig genomics for biomedicine. Nat Biotechnol 31, 122–124 (2013). 23392511 10.1038/nbt.2490

[b2] Prather R. S., Lorson M., Ross J. W., Whyte J. J. & Walters E. Genetically engineered pig models for human diseases. Annu Rev Anim Biosci 1, 203–219 (2013). 25387017 10.1146/annurev-animal-031412-103715 PMC4460601

[b3] Uchida M. *et al.* Production of transgenic miniature pigs by pronuclear microinjection. Transgenic Res 10, 577–582 (2001). 11817545 10.1023/a:1013059917280

[b4] Lavitrano M. *et al.* Efficient production by sperm-mediated gene transfer of human decay accelerating factor (hDAF) transgenic pigs for xenotransplantation. Proc Natl Acad Sci USA 99, 14230–14235 (2002). 12393815 10.1073/pnas.222550299 PMC137866

[b5] Lavitrano M. *et al.* Sperm-mediated gene transfer: production of pigs transgenic for a human regulator of complement activation. Transplant Proc 29, 3508–3509 (1997). 9414813 10.1016/s0041-1345(97)00998-6

[b6] Cabot R. A. *et al.* Transgenic pigs produced using *in vitro* matured oocytes infected with a retroviral vector. Anim Biotechnol 12, 205–214 (2001). 11808636 10.1081/ABIO-100108347

[b7] Pereyra-Bonnet F. *et al.* A unique method to produce transgenic embryos in ovine, porcine, feline, bovine and equine species. Reprod Fertil Dev 20, 741–749 (2008). 18842176 10.1071/rd07172

[b8] Brevini T. A., Antonini S., Cillo F., Crestan M. & Gandolfi F. Porcine embryonic stem cells: Facts, challenges and hopes. Theriogenology 68 Suppl 1, S206–213 (2007). 17582486 10.1016/j.theriogenology.2007.05.043

[b9] Keefer C. L., Pant D., Blomberg L. & Talbot N. C. Challenges and prospects for the establishment of embryonic stem cell lines of domesticated ungulates. Anim Reprod Sci 98, 147–168 (2007). 17097839 10.1016/j.anireprosci.2006.10.009

[b10] Cong L. *et al.* Multiplex genome engineering using CRISPR/Cas systems. Science 339, 819–823 (2013). 23287718 10.1126/science.1231143 PMC3795411

[b11] Mali P. *et al.* RNA-guided human genome engineering via Cas9. Science 339, 823–826 (2013). 23287722 10.1126/science.1232033 PMC3712628

[b12] Miller J. C. *et al.* An improved zinc-finger nuclease architecture for highly specific genome editing. Nat Biotechnol 25, 778–785 (2007). 17603475 10.1038/nbt1319

[b13] Miller J. C. *et al.* A TALE nuclease architecture for efficient genome editing. Nat Biotechnol 29, 143–148 (2011). 21179091 10.1038/nbt.1755

[b14] Moscou M. J. & Bogdanove A. J. A simple cipher governs DNA recognition by TAL effectors. Science 326, 1501 (2009). 19933106 10.1126/science.1178817

[b15] Smith J. *et al.* A combinatorial approach to create artificial homing endonucleases cleaving chosen sequences. Nucleic Acids Res 34, e149 (2006). 17130168 10.1093/nar/gkl720 PMC1702487

[b16] Urnov F. D. *et al.* Highly efficient endogenous human gene correction using designed zinc-finger nucleases. Nature 435, 646–651 (2005). 15806097 10.1038/nature03556

[b17] Sapranauskas R. *et al.* The Streptococcus thermophilus CRISPR/Cas system provides immunity in Escherichia coli. Nucleic Acids Res 39, 9275–9282 (2011). 21813460 10.1093/nar/gkr606 PMC3241640

[b18] Jinek M. *et al.* A programmable dual-RNA-guided DNA endonuclease in adaptive bacterial immunity. Science 337, 816–821 (2012). 22745249 10.1126/science.1225829 PMC6286148

[b19] Wang H. *et al.* One-step generation of mice carrying mutations in multiple genes by CRISPR/Cas-mediated genome engineering. Cell 153, 910–918 (2013). 23643243 10.1016/j.cell.2013.04.025 PMC3969854

[b20] Li W., Teng F., Li T. & Zhou Q. Simultaneous generation and germline transmission of multiple gene mutations in rat using CRISPR-Cas systems. Nat Biotechnol 31, 684–686 (2013). 23929337 10.1038/nbt.2652

[b21] Jao L. E., Wente S. R. & Chen W. Efficient multiplex biallelic zebrafish genome editing using a CRISPR nuclease system. Proc Natl Acad Sci USA 110, 13904–13909 (2013). 23918387 10.1073/pnas.1308335110 PMC3752207

[b22] Nakayama T. *et al.* Simple and efficient CRISPR/Cas9-mediated targeted mutagenesis in Xenopus tropicalis. Genesis 51, 835–843 (2013). 24123613 10.1002/dvg.22720 PMC3947545

[b23] Yu Z. *et al.* Highly efficient genome modifications mediated by CRISPR/Cas9 in Drosophila. Genetics 195, 289–291 (2013). 23833182 10.1534/genetics.113.153825 PMC3761309

[b24] Niu Y. *et al.* Generation of gene-modified cynomolgus monkey via Cas9/RNA-mediated gene targeting in one-cell embryos. Cell 156, 836–843 (2014). 24486104 10.1016/j.cell.2014.01.027

[b25] Hai T., Teng F., Guo R., Li W. & Zhou Q. One-step generation of knockout pigs by zygote injection of CRISPR/Cas system. Cell Res 24, 372–375 (2014). 24481528 10.1038/cr.2014.11 PMC3945887

[b26] Ni W. *et al.* Efficient gene knockout in goats using CRISPR/Cas9 system. PloS one 9, e106718 (2014). 25188313 10.1371/journal.pone.0106718 PMC4154755

[b27] Whitworth K. M. *et al.* Use of the CRISPR/Cas9 system to produce genetically engineered pigs from *in vitro*-derived oocytes and embryos. Biol Reprod 91, 78 (2014). 25100712 10.1095/biolreprod.114.121723 PMC4435063

[b28] Zhou X. *et al.* Generation of CRISPR/Cas9-mediated gene-targeted pigs via somatic cell nuclear transfer. Cell Mol Life Sci 72, 1175–1184 (2015). 25274063 10.1007/s00018-014-1744-7 PMC11113635

[b29] Doench J. G. *et al.* Rational design of highly active sgRNAs for CRISPR-Cas9-mediated gene inactivation. Nat Biotechnol 32, 1262–1267 (2014). 25184501 10.1038/nbt.3026 PMC4262738

[b30] Fu Y., Sander J. D., Reyon D., Cascio V. M. & Joung J. K. Improving CRISPR-Cas nuclease specificity using truncated guide RNAs. Nat Biotechnol 32, 279–284 (2014). 24463574 10.1038/nbt.2808 PMC3988262

[b31] Koike-Yusa H., Li Y., Tan E. P., Velasco-Herrera Mdel C. & Yusa K. Genome-wide recessive genetic screening in mammalian cells with a lentiviral CRISPR-guide RNA library. Nat Biotechnol 32, 267–273 (2014). 24535568 10.1038/nbt.2800

[b32] Port F., Chen H. M., Lee T. & Bullock S. L. Optimized CRISPR/Cas tools for efficient germline and somatic genome engineering in Drosophila. Proc Natl Acad Sci USA 111, E2967–2976 (2014). 25002478 10.1073/pnas.1405500111 PMC4115528

[b33] Levy C., Khaled M. & Fisher D. E. MITF: master regulator of melanocyte development and melanoma oncogene. Trends Mol Med 12, 406–414 (2006). 16899407 10.1016/j.molmed.2006.07.008

[b34] Pingault V. *et al.* Review and update of mutations causing Waardenburg syndrome. Hum Mutat 31, 391–406 (2010). 20127975 10.1002/humu.21211

[b35] Grill C. *et al.* MITF mutations associated with pigment deficiency syndromes and melanoma have different effects on protein function. Hum Mol Genet 22, 4357–4367 (2013). 23787126 10.1093/hmg/ddt285 PMC3888191

[b36] Inui M. *et al.* Rapid generation of mouse models with defined point mutations by the CRISPR/Cas9 system. Sci Rep 4, 5396 (2014). 24953798 10.1038/srep05396 PMC4066261

[b37] Kuscu C., Arslan S., Singh R., Thorpe J. & Adli M. Genome-wide analysis reveals characteristics of off-target sites bound by the Cas9 endonuclease. Nat Biotechnol 32, 677–683 (2014). 24837660 10.1038/nbt.2916

[b38] Wu X. *et al.* Genome-wide binding of the CRISPR endonuclease Cas9 in mammalian cells. Nat Biotechnol 32, 670–676 (2014). 24752079 10.1038/nbt.2889 PMC4145672

[b39] Sakurai T., Watanabe S., Kamiyoshi A., Sato M. & Shindo T. A single blastocyst assay optimized for detecting CRISPR/Cas9 system-induced indel mutations in mice. BMC Biotechnol 14, 69 (2014). 25042988 10.1186/1472-6750-14-69 PMC4118159

[b40] Zhang J. H. *et al.* Improving the specificity and efficacy of CRISPR/CAS9 and gRNA through target specific DNA reporter. J Biotechnol 189C, 1–8 (2014). 10.1016/j.jbiotec.2014.08.033 PMC4252756 25193712

[b41] Zhou J. *et al.* Dual sgRNAs facilitate CRISPR/Cas9-mediated mouse genome targeting. FEBS J 281, 1717–1725 (2014). 24494965 10.1111/febs.12735

[b42] Cui X. S., Li X. Y. & Kim N. H. Global gene transcription patterns in *in vitro*-cultured fertilized embryos and diploid and haploid murine parthenotes. Biochem Biophys Res Commun 352, 709–715 (2007). 17141201 10.1016/j.bbrc.2006.11.092

[b43] Deshmukh R. S. *et al.* DNA methylation in porcine preimplantation embryos developed *in vivo* and produced by *in vitro* fertilization, parthenogenetic activation and somatic cell nuclear transfer. Epigenetics 6, 177–187 (2011). 20935454 10.4161/epi.6.2.13519

[b44] Kono T. *et al.* Birth of parthenogenetic mice that can develop to adulthood. Nature 428, 860–864 (2004). 15103378 10.1038/nature02402

[b45] Wang S., Sengel C., Emerson M. M. & Cepko C. L. A gene regulatory network controls the binary fate decision of rod and bipolar cells in the vertebrate retina. Dev Cell 30, 513–527 (2014). 25155555 10.1016/j.devcel.2014.07.018 PMC4304698

[b46] Yao J. *et al.* Efficient bi-allelic gene knockout and site-specific knock-in mediated by TALENs in pigs. Sci Rep 4, 6926 (2014). 25370805 10.1038/srep06926 PMC4220281

