# Supplementary material for: Efficient CRISPR/Cas9-mediated biallelic gene disruption and site-specific knockin after rapid selection of highly active sgRNAs in pigs
Source: Sci Rep. 2015 Aug 21;5:13348. doi: 10.1038/srep13348 (PMC4543986; doi:10.1038/srep13348)
Supplement: Supplementary Information [file srep13348-s1.doc]

**Supplementary information**

# Efficient CRISPR/Cas9-mediated biallelic gene disruption and site-specific knockin after rapid selection of highly active sgRNAs in pigs

# Xianlong Wang^1,5^, Jinwei Zhou^2,5^, Chunwei Cao^1,5^, Jiaojiao Huang^1,3^, Tang Hai^1^, Yanfang Wang^4^, Qiantao Zheng^1,3^, Hongyong Zhang^1,3^, Guosong Qin^1^, Xiangnan Miao^1^, Hongmei Wang^1,3^, Suizhong Cao^2,*^, Qi Zhou^1,3,*^, Jianguo Zhao^1,3,*^

^1^State Key Laboratory of Reproductive Biology, Institute of Zoology, Chinese Academy of Sciences, Beijing 100101, China

^2^College of Veterinary Medicine, Sichuan Agriculture University, Ya’an, Sichuan 625014, China

^3^University of Chinese Academy of Sciences, Beijing 100049, China

^4^ Institute of Animal Sciences, Chinese Academy of Agricultural Sciences, Beijing 100193, China

^5^These authors contributed equally to this work.

^*^ Corresponding author: J Zhao, [zhaojg@ioz.ac.cn](mailto:zhaojg@ioz.ac.cn); Q Zhou, [qzhou@ioz.ac.cn](mailto:qzhou@ioz.ac.cn); S Cao, [suizhongcao@126.com](mailto:suizhongcao@126.com).

Contents:

1. Supplementary Figure S1
2. Supplementary Table S1
3. Supplementary Table S2

Figure S1 PCR amplification and Sanger sequencing of potential off-target sites.


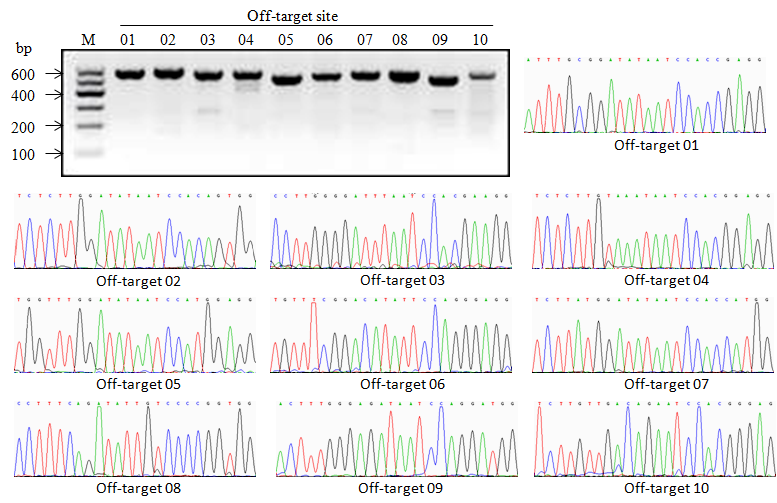


Table S1 Oligonucleotides used in this study.

| **Oligonucleotides for genotyping** | |
| --- | --- |
| Primer name | Sequence |
| Mitf Ex8-F | TCCCTCTCAAGGTGCTCGTC |
| Mitf Ex8-R | AGTCAACTCCCTCATGGCTCA |
| **Oligonucleotides for in vitro transcription** | |
| Primer name | Sequence |
| F1 sgRNA F | TAATACGACTCACTATAGGAACCATTTTAAAAGCCTCCGGTTTTAGAGCTAGAAATAGC |
| F2 sgRNA F | TAATACGACTCACTATAGGTTATATCCGAAAGTTGCAACGTTTTAGAGCTAGAAATAGC |
| R1 sgRNA F | TAATACGACTCACTATAGGCTTTCGGATATAATCCACGGGTTTTAGAGCTAGAAATAGC |
| R2 sgRNA F | TAATACGACTCACTATAGGATCCACGGAGGCTTTTAAAAGTTTTAGAGCTAGAAATAGC |
| sgRNA R | AGCACCGACTCGGTGCCACT |
| **Oligonucleotides for site-specific insertion** | |
| Primer name | Sequence |
| ssODN | CAAGGGAACCATTTTAAAAGCCTCCGGGTACCTGGATTATATCCGAAAGTTGCAACGGG |
| **Oligonucleotides for off-target analysis** | |
| Primer name | Sequence |
| off-target 01F | TAGGCGAGGAGGGAAAATCC |
| off-target 01R | AGCTCCCGTGGCATATAGAG |
| off-target 02 F | ACCCACACTGTTTTCTTGGG |
| off-target 02 R | GATGGCTGGGGTGGTACTTA |
| off-target 03 F | CAGGTGGCTTTTCCATGACC |
| off-target 03 R | CCTCGAATGTTCGTAGCCCA |
| off-target 04 F | ACTGTAGCAGCTAGGGTTGC |
| off-target 04 R | GCCTTCGGAACTAGGATGGA |
| off-target 05 F | TTGGCACCACAGGTATGCTA |
| off-target 05 R | GGGAACTCCCCCATATCATGT |
| off-target 06 F | TGATTGATTCTTGCTGGCGG |
| off-target 06 R | AGGAGCAAGCTCATCTGTCA |
| off-target 07 F | CCCCTGGTCTATATCCCTGC |
| off-target 07 R | CTGTGAGCTGTGGTACATGC |
| off-target 08 F | CACCAGATTTGTCACCACGG |
| off-target 08 R | ATCCCAGAGGCAGATCACAC |
| off-target 09 F | TGCAGAGTTTCCTGCTTTACC |
| off-target 09 R | GCACAGGTGTCTCTACAGGA |
| off-target 10 F | GACCAAATCTGTGCCTCTGC |
| off-target 10 R | CTCCATGCACCTTCACACAG |

Table S2 Potential off-target sites of R1 gRNA.

| **Name** | **Sequence** | **Mismatches (MM)** | **Locus** |
| --- | --- | --- | --- |
| WT | CTTTCGGATATAATCCACGGAGG |  |  |
| Off-target1 | TTTGCGGATATAATCCACCGAGG | 3 MMs [1:4:19] | chrX:+47951260 |
| Off-target2 | CTCTTGGATATAATCCACAGTGG | 3 MMs [3:5:19] | chr1:-170753909 |
| Off-target3 | CTTGGGGATTTAATCCACGAAGG | 4 MMs [4:5:10:20] | chr18:-3069895 |
| Off-target4 | CTCTTGTAAATAATCCACGGAGG | 4 MMs [3:5:7:9] | chrX:+29196411 |
| Off-target5 | GGTTTGGATATAATCCATGGAGG | 4 MMs [1:2:5:18] | chr10:+34908648 |
| Off-target6 | GTTTCGGACATATTCCAGGGAGG | 4 MMs [1:9:13:18] | chr14:-82926750 |
| Off-target7 | CTTATGGATATAATCCACCATGG | 4 MMs [4:5:19:20] | chr6:-141899277 |
| Off-target8 | CTTTCAGATATTGTCCCCGGTGG | 4 MMs [6:12:13:17] | chr1:+7688960 |
| Off-target9 | CTTTGGGAGATAATCCAGGATGG | 4 MMs [5:9:18:20] | chr11:+37552983 |
| Off-target10 | CTTGTTGATAGAATCCACGGGAG | 4 MMs [4:5:6:11] | chr5:+94765245 |
